# Infection by *Parorchis acanthus* (Trematoda) decreases grazing by the keystone gastropod, *Littoraria irrorata*

**DOI:** 10.7717/peerj.4544

**Published:** 2018-03-27

**Authors:** Joseph P. Morton

**Affiliations:** Division of Marine Science and Conservation, Nicholas School of the Environment, Duke University, Beaufort, NC, United States of America

**Keywords:** Grazing, Parasitism, Salt marsh, Behavior modification, Trematode

## Abstract

Parasites are well-known to alter the behavior of their hosts, but there is still a paucity of knowledge about how parasites modify the behavior of many ecologically influential host species. I studied the keystone grazer, the salt marsh periwinkle* (Littoraria irrorata),* to determine the influence of infection by the digenetic trematode, *Parorchis acanthus,* on its grazing behavior. Comparative laboratory grazing studies of wild-collected and experimentally infected snails revealed that *Parorchis* decreased grazing on live *Spartina* by more than 80%. Because of the large ecological influence of *Littoraria* in southern U.S. marshes, parasite modification of snail grazing may have ramifications for marsh ecosystem stability if parasite prevalence is sufficiently high.

## Introduction

A broad body of research demonstrates the ability of parasites to induce powerful changes in the behavior of their hosts (Holmes & Bethel, 1972; Dobson, 1988; Moore & Gotelli, 1990; Lafferty & Morris, 1996; Moore, 2002; Toscano, Newsome & Griffen, 2014; Soghigian, Valsdottir & Livdahl, 2017). These changes may be adaptive for the parasite because behavioral modification often facilities transmission to the next host species in its life cycle, an adaptive response of the host species, or a non-adaptive byproduct of parasitic infection (Lafferty, 1999; Levri, 1999; Moore, 2002). Such behavioral changes can vary widely in their magnitude (Poulin, 2010; Moore, 2002). In one well-known example, a digenetic trematode *Dicrocoelium dendriticum*, manipulates its ant intermediate host into climbing up and clinging to the tip of a grass blade where it waits to be transmitted to its sheep final host via accidental consumption (Carney, 1969; Moore, 2002). In most instances, however, behavioral changes are rarely this spectacular and are more often manifested as subtle shifts in the frequency of a particular activity like feeding or movement (Moore, 2002; Lefèvre et al., 2009; Poulin, 2010; Blakeslee et al., 2015). There now exists a growing body of evidence that demonstrates the ability of parasites to indirectly influence ecosystem structure, functioning, and dynamics through modifying the behavior of numerically dominant or otherwise ecologically important host species (Thomas et al., 1998; Mouritsen & Poulin, 2005; Wood et al., 2007; Hernandez & Sukhdeo, 2008; Sato et al., 2012; Sánchez et al., 2016). Despite this, there is still very little known about how parasites modify the behavior of many influential host species.

In aquatic systems, gastropods serve as the intermediate hosts for many species of digenetic trematode parasites (Lauckner, 1987; Mouritsen & Poulin, 2002) that are well-known to induce a wide range of behavioral changes, including consumption rate (Mouritsen & Jensen, 1994; Wood et al., 2007; Clausen et al., 2008), predator avoidance (Bernot, 2003; Kamiya & Poulin, 2012; Swartz et al., 2015), microhabitat selection (McCurdy, Boates & Forbes, 2000; Curtis, 2007; O’Dwyer, Kamiya & Poulin, 2014) and movement (Curtis, 1990; Belgrad & Smith, 2014; O’Dwyer, Kamiya & Poulin, 2014). Because many gastropod species comprise a large portion of ecosystem free-living biomass and can regulate ecosystems through activities like grazing, trematode-induced behavior modifications have the potential to indirectly affect ecosystem structure and function (Wood et al., 2007; Kuris et al., 2008). Despite the ubiquity of trematode-snail associations, there is still a paucity of information about the effects of trematodes on many gastropod hosts’ behaviors, even ecologically influential host species.

In southern US marshes, the abundant marsh periwinkle, *Littoraria irrorata*, climbs stems of the foundational marsh cordgrass, *Spartina alterniflora*, with the rising tide to avoid foraging predators (Hamilton, 1976; Hamilton, 1978; Vaughn & Fisher, 1988). While on plant stems, these snails participate in a facultative, proto-farming mutualism by producing longitudinal grazing scars on plant leaves with their radula that are subsequently invaded by intertidal fungal pathogens which snails consume (Silliman & Newell, 2003). Fungal removal experiments have demonstrated that snail grazing acts synergistically with fungal pathogens to exert top-down suppression of salt marsh productivity (Silliman & Newell, 2003). *Littoraria* also acts as the first intermediate host for at least five species of digenetic trematode (Holliman, 1961; Coil & Heard , 1966; Heard, 1968; Heard, 1970). One of its commonly occurring trematodes, *Parorchis acanthus* (Nicoll, 1907), uses several marine and estuarine gastropods as first intermediate hosts, encysts as metacercariae externally on hard substrate (e.g., crab carapaces), and reaches sexual maturity in the cloaca and the Bursa of Fabricius of many species of shorebirds (mainly Charadriiformes) after metacercariae are trophically transmitted via shorebird consumption (Stunkard & Cable, 1932; Holliman, 1961; Cooley, 1962; Ching, 1978; Dronen & Blend, 2008; Diaz, Cremonte & Navone, 2011). While both grazing behavior and ecological influence of *Littoraria* have been well-studied (Graça, Newell & Kneib, 2000; Silliman & Zieman, 2001; Silliman & Bertness, 2002; Silliman & Newell, 2003; Silliman et al., 2005) no work has yet investigated the consequences of trematode parasitism on snail grazing in this species.

Here, I conducted laboratory grazing assays to investigate the impact of *Parorchis* infection on the consumption of live *Spartina* tissue by *Littoraria*. I collected and identified infected and uninfected *Littoraria* from the field and evaluated differences in grazing rate in a laboratory experiment. I conducted another grazing assay in which I evaluated differences in grazing rate before and after experimentally infecting *Littoraria* with *Parorchis* to establish that differences in grazing resulted from infection with *Parorchis* and not pre-existing snail characteristics that predisposed them to becoming infected. Based on field and laboratory behavioral observations of *Littoraria* infected with *Parorchis,* I predicted that infection might diminish grazing rate. This study is the first to systematically investigate the impact of trematode parasitism on a key behavior of this abundant and ecologically influential gastropod.

## Materials and Methods

To determine the effects of parasite infection on snail grazing of live *Spartina*, I first employed a laboratory grazing assay using field-collected *Littoraria* whose infection status was determined via cercarial shedding. All snails and *Spartina* used in this experiment were collected from the Hoop Pole Creek Clean Water Reserve in Atlantic Beach, North Carolina, USA (North Carolina Coastal Federation permit #HPC-104).

Snails were periodically collected at low tide from May to June, 2013. The particular marsh zone from which snails were collected was noted for each collection—tall *Spartina* zone (*n* = 3 collections), intermediate *Spartina* zone (*n* = 7 collections), and snail front associated with marsh die-off borders (*n* = 15 collections). Before snails were evaluated for infection with trematodes, they were kept in a dry 5 gallon bucket with a lid for 24–48 h. Keeping snails in dry conditions for this period guaranteed that any infected individuals would always shed cercariae when submerged in seawater. After the drying period, collected snails were gently washed in filtered seawater and placed in 60 × 15 mm petri dishes. Each dish was filled entirely with filtered seawater (∼30 mL) and sealed with a plastic lid such that the snail was completely immersed and unable to escape. Petri dishes with snails were placed in a 17.2 × 10.6 × 6.3 inch aquarium where they were allowed to sit for 6 h under a 250-W heat lamp to encourage shedding (Koprivnikar & Poulin, 2009). After this period, the contents of each dish were inspected under a stereo microscope to determine the presence of shed cercariae. For each infected individual, cercariae were identified to species level using published keys (Gibson, Jones & Bray, 2008). Only snails infected with *Parorchis* were used in experiments. All snails examined in this way (including those used in experiments) were ultimately dissected at the end of the study to confirm the efficacy of this identification method. This method yielded 0% false negatives.

Ungrazed *Spartina* leaves used in the experiment were taken from a 0.25 m^2^ patch within an intermediate marsh area where snail recruitment is very low and grazing adults are absent year-round. After collection, leaves were rinsed under running tap-water to remove any dirt or epiphytes. Each leaf was cut at the ligule, the basal 5 cm portion of the blade was discarded, and the adjacent 5 cm section was used in the experiment. After the width of each 5 cm leaf section was measured, the leaf was folded in half lengthwise and cut into twin pieces which were weighed separately. One member from each set of twin leaf sections was used to evaluate snail grazing while the other leaf segment served as a control (Graça, Newell & Kneib, 2000).

Glass bottles with perforated plastic lids served as experimental units in which *Spartina* and *Littoraria* were established. All bottles contained a leaf section and 2 ml of artificial seawater (30‰). Each bottle was assigned to one of two treatments (*n* = 60 individuals/treatment): one snail infected with *Parorchis* or one uninfected control. Bottles without snails served as controls for natural reduction of *Spartina* biomass in the absence of snails (*n* = 15 individuals/treatment).

Snails were starved for 24 h before being placed in experimental bottles. Snails were then allowed to consume live *Spartina* for 72 h at which time leaf fragments were removed from bottles and gently washed to remove mucus and snail fecal pellets. The total length of grazing wounds on each leaf segment was measured before leaves were dried to a constant weight in an oven at 50 °C. After drying, leaf biomass was determined to the nearest 0.00001 g. Shell lengths of all snails were measured with calipers to the nearest 0.01 cm. Because larger snails are likely to consume more than smaller snails, dry biomass of snail tissues was determined so that this factor could be evaluated for its influence on consumption (Graça, Newell & Kneib, 2000; Atkins et al., 2015). After cracking the shell with a hammer, snail soft tissues were extracted and placed into numbered, preweighed, aluminum tins. Tins with snail tissues were dried to a constant weight in an oven at 50 °C (∼2 wks). Consumption was calculated as the difference in dry biomass between control and grazed leaf sections. Differences in consumption were evaluated in R (R Core Team, 2014) using a general linear model (GLM) with the main effect infection status and the covariates snail length, snail dry biomass, snail sex, initial leaf wet weight and leaf width. Data that were heteroscedastistic or not normally distributed were square root transformed. After transformation, visual inspections of residual plots did not reveal any obvious deviations from homoscedasticity or normality.

To ensure that observed differences in snail grazing behavior were the result of infection with *Parorchis* and not inherent behavioral differences that increased infection susceptibility (e.g., snails that naturally consume less could potentially have weakened defenses against parasitic infection), I collected 50 snails from a marsh area with low infection prevalence, confirmed that they were uninfected through the proven cercariae shedding method, assigned them individual numbers, evaluated grazing using the aforementioned methodology, and then randomly selected half of those snails to be experimentally infected with *Parorchis.*

To infect snails, adult *Parorchis* were isolated from the bursa fabricii and cloaca of clapper rails (*Rallus longirostrus*) donated by local hunters in November, 2014. Live miracidia were obtained by extracting eggs from the uterus of adult flukes that had been placed in a dish of filtered seawater. Under a dissecting microscope, eggs were observed hatching almost immediately upon contact with the seawater. Individual miracidia were then pipetted into numbered 60 × 15 mm petri dishes containing filtered seawater and a single, uninfected snail. Lids were placed on dishes and snails were exposed to miracidia for 36 h. The remaining 25 uninfected snails were placed in covered dished containing no miracidia as a procedural control. After the exposure period, snails were removed from their dishes and placed in 16 oz glass mason jars labeled with the number corresponding to their exposure dish and filled with ∼2.5 oz of seawater. To allow adequate air supply, jars were sealed with a square piece of window screen mesh, secured to the jar’s opening with a rubber band. Snails were maintained in these jars for 16 weeks, during which time they were given dead *Spartina* stems covered in fungus for food. Both the food supply and water within jars was changed weekly.

After the 16 wk period, snails were evaluated for infection via isolation and cercarial shedding. Of the 25 individuals exposed to miracidia, six died, four showed no sign of infection, and 15 shed cercariae of *Parorchis*. Subsequent dissection of the six snails in the experimental infection treatment group that died revealed that they had been successfully infected. Of the 25 control snails, one died and the remaining 24 shed no cercariae. The remaining 15 infected snails and 15 randomly selected control snails were subjected to the grazing assay again following the same methodology.

To test for differences between initial and final grazing intensity and consumption rate between experimentally infected snails and uninfected controls, I used the lmerTest package (Kuznetsova, Brockhoff & Christensen, 2017) in R to perform a generalized linear mixed effects analysis (GLMM) with the main effects being treatment group (experimentally infected or control), time (before or after experimental treatment applied), final infection status, and covariates snail length, snail dry biomass, snail sex, initial leaf wet weight and width, and bottle as a random effect. Tukey’s Honest Significant Difference tests, calculated with the glht function from the multcomp library, were used for post-hoc analysis when necessary (Hothorn, Bretz & Westfall, 2008). Differences in mortality between treatment groups were analyzed with a Chi-squared test of independence. As in the previous experiment, any data that deviated from normality or homogeneity of variance were square root transformed such that they met these assumptions.

## Results

Average infection prevalence of *Parorchis* from all 25 field collections of *Littoraria* used in the initial grazing assay was 7.68% ± 1.56 SE. Average prevalence around die-off borders was high (11.31% ± 2.1 SE) compared to healthy intermediate *Spartina* areas (3.25% ± 0.51 SE) and tall *Spartina* areas associated with the marsh edge (0.76% ± 0.39 SE). The vast majority of infected snails collected from the field shed the cercariae of *Parorchis* (97.7%). Only six snails of the 3,616 snails collected were infected with other trematode species (five were infected with *Levinseniella carteretensis* and one with an unidentified avian blood fluke). No double infections were observed.

**Figure 1:**
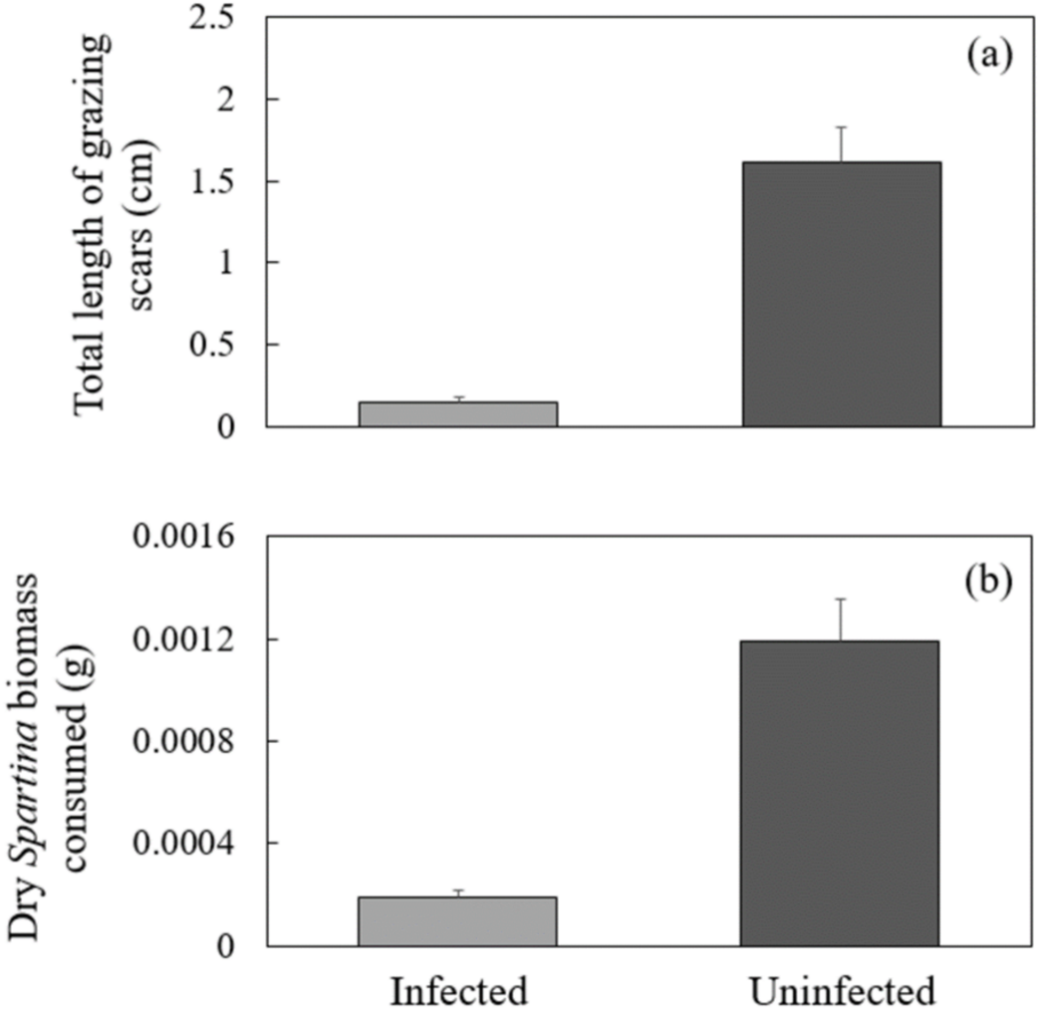
Comparisons of (A) total length of grazing scars and (B) consumption rate of *Littoraria* naturally infected with *Parorchis* and uninfected snails. Data are means and SE; *n* = 60 individuals/treatment.

In my initial grazing assay using wild-collected infected and uninfected snails it was not necessary to correct for changes in biomass of initial leaf segments since there was no significant change in the biomass of control leaves over the 72 h experimental period (Paired *t*-test, *P* > 0.17). Snail sex, snail size (shell length), and initial leaf width and wet biomass were not significant predictors of either consumption rate or grazing scar length (general linear model, *P* > 0.05, all cases). The total length of grazing scars produced by infected snails was significantly less than their uninfected counterparts (Fig. 1A, GLM, *P* < 0.0002). Diminished intensity of grazing by infected snails translated to significant reductions in live *Spartina* biomass of >80% compared to uninfected controls (Fig. 1B, GLM, *P* < 0.008).

**Figure 2:**
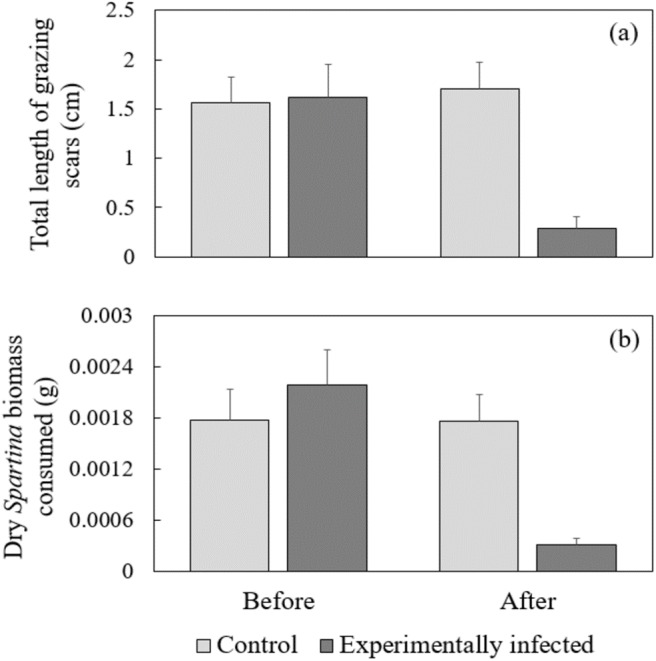
Comparisons of (A) total length of grazing scars and (B) consumption rate of *Littoraira* before and after being experimentally infected with *Parorchis* and uninfected controls. Data are means and SE; *n* = 15 individuals/treatment.

In my grazing study using experimentally infected snails, snail length, snail dry biomass, snail sex, initial leaf wet weight, and leaf width were not significant predictors of either consumption rate or grazing scar length (GLMM, *P* > 0.14, all cases). A significant interaction between time and treatment provided strong evidence of the impact of experimental infection on both consumption rate and grazing scar length (general linear mixed effects model, *P* < 0.007, both cases). Prior to experimental infection of snails the total length of grazing scars and the reduction in *Spartina* biomass produced by treatment groups were not significantly different (Tukey HSD, *P* > 0.1, both cases). After 16 wks, snails experimentally infected with *Parorchis* were associated with significantly reduced grazing scar length (Fig. 2A, Tukey HSD, *P* < 3.8e^−5^) as well as significantly diminished reductions in *Spartina* biomass compared to before being infected (Fig. 2B, Tukey HSD, *P* < 0.00017). There was no significant difference in grazing scar length or *Spartina* dry biomass observed in the control group before and after the 16 wk period (Figs. 2A and 2B, Tukey HSD, *P* > 0.5, both cases). Comparisons between treatment groups after the experiment yielded results similar to the initial assay using naturally-infected individuals. Mean consumption rate and length of grazing scars produced by infected snails were significantly less than uninfected controls (Tukey HSD, *P* < 0.0099, both cases). Mortality rate of snails exposed to miracidia of *Parorchis* was significantly higher than unexposed controls (Chi-squared test of independence, *χ*^2^ = 0.415, *P* > 0.05).

## Discussion

Combined, my comparative laboratory studies and experimental manipulations demonstrate that infection with the trematode, *Parorchis acanthus,* can have a powerful negative impact on the ability of *Littoraria irrorata* to graze live *Spartina alterniflora* tissue. Reduced grazing behavior by experimentally infected snails confirms that these observed behavioral changes in *Littoraria* are the result of infection and not an inherent characteristic of the gastropod host that increases predisposition to infection. As marsh periwinkles are keystone grazers in ecologically and economically important southern salt marshes, these laboratory results call on future studies to focus on determining if these parasites can serve as important indirect determinants of marsh community structure in the field.

While the effects of *Parorchis* on *Littoraria* grazing were apparent, I did not directly investigate the possible mechanisms underlying the reduction in grazing intensity. In many gastropod species, the asexual multiplication of larval trematodes in gastropod tissues yields serious injury and pathological changes that can potentially drive changes in host energetics, metabolism, and feeding behavior (Lauckner & Kinne, 1980). For instance, infestation with larval trematodes in the intertidal snail, *Littorina littorea* yields varying degrees of damage to the gonad—digestive gland complex, resulting in a general decrease in host consumption rate (Lauckner & Kinne, 1980; Wood et al., 2007; Clausen et al., 2008; Larsen & Mouritsen, 2009). The magnitude of tissue destruction and subsequent behavioral changes may vary with both the intensity of infestation and the characteristics of the particular parasite species involved (Lauckner & Kinne, 1980; Curtis, 1985; Levri & Lively, 1996; McCurdy, Boates & Forbes, 2000; Wood et al., 2007; Clausen et al., 2008). In *Littoraria*, several possible mechanisms could underlie observed changes to grazing. In *Littoraria* with mature infections, the rediae of *Parorchis* extend throughout the visceral mass, potentially compromising the digestive gland which could limit digestive efficiency or the capacity to feed (Lauckner & Kinne, 1980; Lauckner, 1987; Wood et al., 2007; Clausen et al., 2008). Moreover, reduced energetic demands as a result of parasitic castration could be the driving or co-occurring mechanism that leads to a reduction in grazing (Lauckner & Kinne, 1980; Wood et al., 2007; Clausen et al., 2008; Larsen & Mouritsen, 2009). Further work on the pathology of infection with *Parorchis* in *Littoraria* is required for a mechanistic understanding of behavior modification.

I did not quantify the effects of infection by other trematode parasite species on *Littoraria* grazing because of their comparative rarity in collections (<3% of all infected snails). Whether or not these species can be found in abundance at other marsh sites and whether or not they too have similar effects on snail grazing is the subject of future work. Additionally, previous work on trematode parasitism in another marsh-dwelling gastropod, *Certhidea scalariformis* found that infection with *Parorchis acanthus* disrupted circatidal climbing behavior, causing infected snails to migrate less and remain closer to the marsh surface at low tide (Belgrad & Smith, 2014). Given these results and given the ecological significance of circatidal climbing in *Littoraria* as an antipredator behavior (Hamilton, 1976; Hamilton, 1978), future studies should examine the effects of *Parorchis* on climbing in *Littoraria*. Moreover, because there is some evidence to suggest that *Parorchis acanthus* may represent a cryptic taxa containing several species, increased clarification of this taxa may be necessary to more fully understand its ecology (Huspeni, 2000; Dronen & Blend, 2008).

Because *Littoraria* is known to strongly suppress the growth of *Spartina* (Silliman & Zieman, 2001; Silliman & Bertness, 2002)*,* and drive marsh die-off at high densities (Silliman et al., 2005), parasitic modification of snail grazing could have consequences for marsh structure and function when infection prevalence is sufficiently high. In this study, I found that infection prevalence with *Parorchis* from all field collections was fairly low (median prevalence was 5%). However, prevalence was elevated in the snail consumer fronts associated with marsh die-off areas, in some cases exceeding 30%. This difference may be due to variation in bird density—the final hosts of *Parorchis*. It is generally acknowledged that the distribution of final hosts governs larval trematode recruitment to snails and a wide body of research that has shown positive correlations between the prevalence of larval trematodes in gastropod hosts and the abundance of avian final hosts (Robson & Williams, 1970; Bustnes & Galaktionov, 1999; Skirnisson, Galaktionov & Kozminsky, 2004; Hechinger & Lafferty, 2005; Byers et al., 2008). I would predict a high level of bird usage in marsh die-off areas where infection prevalence of *Parorchis* is relatively high.

While I did not explore the ramifications of parasite-induced modification of grazing for salt marsh community organization, dramatic reductions in grazing observed in infected individuals in addition to observations of locally high field infection prevalence suggest that such changes could yield non-trivial, local impacts. If one assumes that parasites reduce individual snail grazing intensity by 80%, based on this laboratory study, then a 30% infection prevalence (the naturally-occurring maximum prevalence observed in field collections) would reduce the impact of snails in an area by approximately 24%. This would likely translate to strong impacts on community structure in areas with moderate snails densities where their top-down effects just start to emerge (60–144 ind per m^2^) (Silliman & Zieman, 2001; Silliman & Bertness, 2002). Below that density, snails do not intensively graze live grass, feeding instead on abundant dead organic matter. If parasites push the effective impact of snails below this critical density threshold they are likely to dampen top-down effects. At high densities (300–1,000 snails/m^2^), where snails form consumer fronts that yield cascading vegetation loss, a reduction of grazing impacts by 25% would likely reduce the rate of ecosystem loss but not halt it entirely. Reversing this loss would necessitate parasites moving snail impacts below die-off thresholds that have been established (Silliman & Zieman, 2001; Silliman & Bertness, 2002; Atkins et al., 2015). Additionally, I observed higher rates of mortality in experimentally infected snails in the lab, indicating the potential role of parasites in mediating lethal effects. If similar patterns of differential mortality occur in the field, this effect of parasitism could also have ramifications for marsh structure by reducing snail densities. Experimental manipulation of parasite prevalence in the field is necessary to determine whether these laboratory results translate to real-world effects on marsh communities.

##  Supplemental Information

10.7717/peerj.4544/supp-1 Supplemental Information 1 Raw data from all grazing experiments and field collections

## Additional Information and Declarations

### Field Study Permissions

The following information was supplied relating to field study approvals (i.e., approving body and any reference numbers): Field collections at the Hoop Pole Creek Clean Water Reserve were authorized by a permit from the North Carolina Coastal Federation issued to Rachel Gittman (project number: HPC-104).

### Data Availability

The following information was supplied regarding data availability: The raw data are included in Supplemental Information 1.
